# Brief Report: Association Between Pharmacologic Tenofovir Adherence Measures and Subsequent 24-Week Viral Load Outcomes for People With HIV in South Africa

**DOI:** 10.1097/QAI.0000000000003866

**Published:** 2026-03-26

**Authors:** Nicola Bodley, Katya Govender, Pravikrishnen Moodley, Natasha Samsunder, Yukteshwar Sookrajh, Philip J. Turner, Christopher C. Butler, Gail N. Hayward, Monica Gandhi, Paul K. Drain, Nigel Garrett, Jienchi Dorward

**Affiliations:** a Centre for the AIDS Programme of Research in South Africa (CAPRISA), University of KwaZulu–Natal, Durban, South Africa;; b Africa Health Research Institute, Durban, South Africa;; c Department of Virology, University of KwaZulu-Natal and National Health Laboratory Service, Inkosi Albert Luthuli Central Hospital, KwaZulu-Natal, South Africa;; d eThekwini Municipality Health Unit, Durban, South Africa;; e Nuffield Department of Primary Care Health Sciences, University of Oxford, Oxford, United Kingdom;; f NIHR HealthTech Research Centre in Community Healthcare, Oxford, United Kingdom;; g Division of HIV, Infectious Disease, and Global Medicine, Department of Medicine, University of California, San Francisco (UCSF), San Francisco, CA;; h Department of Global Health, Schools of Medicine and Public Health, University of Washington, Seattle, WA;; i Department of Medicine, School of Medicine, University of Washington, Seattle, WA;; j Department of Epidemiology, School of Public Health, University of Washington, Seattle, WA;; k Discipline of Public Health Medicine, School of Nursing and Public Health, University of KwaZulu-Natal, Durban, South Africa; and; l Desmond Tutu HIV Centre, University of Cape Town, Cape Town, South Africa.

**Keywords:** HIV, point-of-care, urine assay, tenofovir, adherence, viral load

## Abstract

**Background:**

Objective measures of antiretroviral therapy adherence, such as tenofovir (TFV) concentrations, may allow for targeted interventions to prevent future HIV viremia. We evaluated 3 TFV adherence measures and their association with current and 24-week viral load outcomes.

**Methods:**

In a South Africa-based trial, we measured urine TFV using a point-of-care (POC) antibody-based assay and 2 metrics assessed through liquid-chromatography-mass-spectrometry: quantitative urine TFV and TFV-diphosphate (TFV-DP) concentrations in dried blood spots (DBS). Logistic regression assessed the association between current and subsequent 24-week viral load outcomes and these 3 measures. We also compared the baseline characteristics of individuals with detectable vs. undetectable POC urine TFV results at enrollment.

**Results:**

Of 124 participants, 54.8% female, median age 39 years, 100 (81.5%) had detectable POC urine TFV and 23 (18.5%) did not. Higher TFV-DP concentrations in DBS were negatively associated with viremia at 24 weeks [odds ratio (OR) 0.83, 95% confidence interval (CI): 0.725–0.928, *P* = 0.003], while a detectable POC urine TFV (OR 0.62, 95% CI: 0.22–1.82, *P* = 0.380) and quantitative urine TFV (OR 0.98, 95% CI: 0.95–1.00, *P* = 0.153) showed no significant association. Compared with those with detectable POC urine TFV, those with undetectable POC urine TFV were more likely to be viremic at enrollment (78.3% vs. 25.7%, *P* < 0.001) and have a CD4 count <200 cells/uL (34.8% vs. 12.9%, *P* = 0.001).

**Conclusions:**

DBS TFV-DP was associated with viremia at 24 weeks and could help predict future viremia. Undetectable POC urine TFV was associated with recent antiretroviral therapy initiation, current viremia, and lower CD4 count.

**Trial Registration:**

Pan African Clinical Trials Registry (PACTR202001785886049) and the South African Clinical Trials Registry (DOH-27-072020-6890).

## INTRODUCTION

Lifelong adherence to antiretroviral therapy (ART) is crucial for people with HIV (PWH), but assessing and supporting adherence can be challenging. Viral load (VL) measurement is the primary marker of treatment outcomes, with an undetectable VL <50 copies/mL indicating treatment success.^1^ However, VL monitoring is costly, cannot differentiate between poor adherence and viral resistance, and relies on centralized laboratories, with delayed results hindering timely interventions. In addition, measuring VL allows detection of established viremia, which may leave little time to intervene before viremia occurs, which can lead to clinical deterioration and transmission to partners. Measuring ART drug concentrations can objectively assess adherence and identify PWH at risk of future treatment failure despite current viral suppression, enabling early intervention before viral suppression is lost.

In South Africa, tenofovir disoproxil fumarate (TDF) continues to be widely used, with current (2023) HIV treatment guidelines recommending a fixed combination of TDF, lamivudine, and dolutegravir for first-line ART.^2^ Dolutegravir is well tolerated, effective in achieving and maintaining viral suppression, and has a high genetic barrier to resistance.^3,4^ The fixed dose combination of TDF, lamivudine, and dolutegravir means that measures of tenofovir (TFV) concentrations may be particularly useful for measuring overall adherence to this widely used single tablet combination.

Both short- and long-term measures of TFV adherence exist. Tenofovir–diphosphate (TFV-DP) concentrations measured in dried blood spots (DBS) by liquid chromatography-tandem mass spectrometry (LC-MS/MS) are associated with TDF exposure in the preceding 6‒8 weeks,^5^ and can predict future viremia, however, this technology still requires samples to be sent to and processed in laboratories.^6–8^ More clinically accessible methods of TFV assessment are needed, such as antibody-based point-of-care (POC) urine TFV assays that provide a measure of recent TDF exposure (preceding 3‒4 days), giving insight into recent or short-term adherence.^9^ POC-TFV assays can provide results within minutes in a clinic setting. Use of POC TFV assays in combination with adherence counseling has already been associated with high levels of resuppression after virologic failure.^10^ Furthermore, POC-TFV, quantitative urine TFV by LC-MS/MS, and TFV-DP are associated with concurrent viremia.^11,12^ However, whether POC-TFV and quantitative urine TFV measures are associated with future viremia has not been demonstrated.

This study aimed to determine whether TFV-DP measurement on DBS, quantitative urine TFV measured in the laboratory, and POC-TFV are associated with subsequent 24-week VL values. It also aimed to compare the baseline characteristics of individuals in whom TFV was and was not detected in the urine (based on the POC-TFV assay).

## METHODS

### Study Design

We conducted a substudy within the POwER trial, a feasibility study comparing POC VL testing with standard laboratory VL testing among people with recent viremia (VL ≥1000 copies/mL), which has been described previously.^13,14^

### Setting

The study took place at an urban and rural clinic in KwaZulu-Natal, South Africa.

### Participants

We included all POwER participants who were taking TDF. PWH were eligible for POwER if they were receiving first-line dolutegravir or efavirenz-based ART and with recent viremia >1000 copies/mL in the past 6 weeks, for which they had not yet received enhanced adherence counseling as recommended by the World Health Organization (WHO).

### Procedures

Consent for this subanalysis was included in the original POwER consent. Baseline demographic, behavioral, and clinical characteristics of participants were collected at enrollment through nurse or counsellor-conducted questionnaires. Enhanced adherence counseling recommended by the South African National Department of Health HIV guidelines was conducted for all participants, and all consenting participants had urine, whole blood, and plasma samples taken and stored at −80°C, for retrospective testing. Participants were randomized to receive either POC or standard laboratory-based repeat VL testing after 12 weeks, with the primary outcome VL measured 24 weeks after enrollment (see Supplement Table S1, Supplemental Digital Content, http://links.lww.com/QAI/C668). The VL measured at enrollment is considered current VL in this study.

### Laboratory Testing

Urine was thawed and sent for quantitative urine TFV adherence testing using LC-MS/MS (Supplement Section 1: Liquid chromatography and dual tandem mass spectrometry (LC-MS/MS) sample analysis protocol) at the Africa Health Research Institute in Durban, and for POC-TFV assay (Abbott Laboratories, Abbott Park, IL) testing at the CAPRISA Clinical Trial Unit Laboratory. The Abbot POC-TFV assay is an antibody-based test for TFV that uses Lateral Flow Immunoassay technology and was previously validated against the reference standard of TFV detection and quantification, LC-MS/MS.^4^ The POC-TFV assay has a threshold (concentration of ≥1500 ng/mL) to determine whether a person has taken at least 1 dose of TDF within the prior 4 days. Specifically, 3–4 urine drops were added to the test well, and the result was read by 2 independent laboratory technicians after 3–5 minutes. Photographs of discrepant results were adjudicated by a third investigator. For TFV-DP measurement, DBS were prepared from EDTA specimens and stored at −80°C before being thawed and analyzed using LC-MS/MS at Africa Health Research Institute. The 24-week VL was measured in thawed plasma samples at the National Health Laboratory Service at the Inkosi Albert Luthuli Hospital in Durban (Supplement Section 2: Details of HIV viral load testing). Urine TFV and DBS TFV-DP are stable when stored at −80°C and the POC-TFV and TFV-DP assays have been validated on frozen samples.^5,6^

### Exposures and Outcomes

For the primary analysis, the exposures of interest were POC-TFV, TFV-DP in DBS, and quantitative urine TFV using LC-MS/MS, with the primary outcome being viremia (VL > 50 copies/mL) at 24 weeks.

In a secondary analysis to describe baseline characteristics associated with a negative POC-TFV result, the exposures of interest were baseline sociodemographic and laboratory (including current VL and CD4 cell count) factors, and the outcome of interest was detectable vs. undetectable urine POC-TFV.

### Statistical Analysis

For the main analysis, we used separate univariable logistic regression models to analyze the relationship between each of the measures of adherence (binary POC-TFV [detected vs. not detected], quantitative urine TFV, and quantitative TFV-DP in DBS as independent exposures) and the outcome of 24-week viremia (VL > 50 copies/mL). We rescaled TFV-DP to every increase of 100 fmol/punch, and urine TFV to every increase of 1000 ng/mL, to aid in interpretation of odds ratios. In a sensitivity analysis, we included an interaction term between the TFV measures and a binary exposure variable of baseline ART regimen (dolutegravir or efavirenz based), to evaluate whether any associations differed by ART regimen. In a post hoc subanalysis restricted to people with baseline undetectable VL <50 copies/mL, we determined the number of people who have “TFV-DP/VL mismatch,” that is, undetectable VL but a low TFV-DP <800 fmol/punch (threshold defined based on findings from previous studies^1,7^), and described the proportion with 24-week viremia, and with undetectable POC urine TFV, in those with and without mismatch.

In a secondary analysis, we described baseline clinical and laboratory characteristics of participants with detectable vs. undetectable TFV in the urine, using the POC TFV assay, and compared the proportions using χ^2^ tests.

### Ethical Approval

The POwER study received approval from the University of KwaZulu-Natal Biomedical Research Ethics Committee (BREC00000836/2019) and the University of Oxford Tropical Research Ethics Committee (OxTREC66-19).

## RESULTS

### Study Population

A total of 124 participants who were taking TDF were enrolled into the POwER study between August 20, 2020, and March 25, 2022. Most were women (68/124, 54.8%) and the median age was 39.0 years [interquartile range (IQR) 34.0–45.0 years, Table 1]. Participants had been on ART a median duration of 4.1 years (IQR 1.5–6.1 years) and had been on their current regimen (40.3% dolutegravir -, 59.7% efavirenz-based regimen) for a median of 1.6 years (IQR 0.7–5.0 years), all had been on TDF-containing ART for ≥3 months before enrollment. Participants presented a median of 15 days (IQR 12.8–21.0 days) since their pre-enrollment detectable VL result, which determined their eligibility for POwER.

**TABLE 1.** Baseline Characteristics of Participants Stratified by Urine TFV Results

Characteristic	Variable	Total	TFV Not Present (n = 23)	TFV Present (n = 101)	*P*
Age, yrs	Median (IQR)	39.0 (34.0–45.0)	38.0 (34.0–51.0)	39.0 (34.0–45.0)	0.938
Gender	Female	68 (54.8)	9 (39.1)	59 (58.4)	0.108
	Male	56 (45.2)	14 (60.9)	42 (41.6)	
Time since ART initiation, yrs	Median (IQR)	4.1 (1.5–6.1)	2.0 (0.7–4.4)	4.3 (2.1–6.2)	0.002
Time on current regimen, yrs	Median (IQR)	1.6 (0.7–5.0)	0.8 (0.5–2.1)	2.1 (0.8–5.2)	0.017
Time on dolutegravir-based regimen, yrs	Median (IQR)	0.6 (0.5–1.0)	0.6 (0.5–1.1)	0.6 (0.5–1.0)	0.807
Current TB	No	122 (98.4)	23 (100.0)	99 (98.0)	1.000
	Yes	2 (1.6)		2 (2.0)	
ART side effects	No	120 (96.8)	23 (100.0)	97 (96.0)	1.000
	Yes	4 (3.2)		4 (4.0)	
Last time participant missed a dose of ART	<2 wk	30 (24.2)	11 (47.8)	19 (18.8)	0.014
	2–4 wk	16 (12.9)	3 (13.0)	13 (12.9)	
	1–3 mo	16 (12.9)	4 (17.4)	12 (11.9)	
	>3 mo	8 (6.5)	1 (4.3)	7 (6.9)	
	Never	54 (43.5)	4 (17.4)	50 (49.5)	
Number of ART doses missed in past 4 d	0	95 (76.6)	11 (47.8)	84 (83.2)	<0.001
	1	14 (11.3)	3 (13.0)	11 (10.9)	
	2	9 (7.3)	5 (21.7)	4 (4.0)	
	3	2 (1.6)	1 (4.3)	1 (1.0)	
	4	4 (3.2)	3 (13.0)	1 (1.0)	
Ethnicity	Black African	121 (97.6)	22 (95.7)	99 (98.0)	0.463
	Colored	1 (0.8)		1 (1.0)	
	Other	2 (1.6)	1 (4.3)	1 (1.0)	
Enrollment CD4 count category, cells/µL	<200	21 (16.9)	8 (34.8)	13 (12.9)	0.001
	200–349	25 (20.2)	7 (30.4)	18 (17.8)	
	350–499	29 (23.4)	6 (26.1)	23 (22.8)	
	≥500	49 (39.5)	2 (8.7)	47 (46.5)	
Time since pre-enrollment viral load, d	Median (IQR)	15.0 (12.8–21.0)	15.0 (13.5–18.0)	14.0 (12.0–21.0)	0.747
ART regimen at enrollment	TDF/3 TC/DTG	49 (39.5)	12 (52.2)	37 (36.6)	0.237
	TDF/FTC/EFV	75 (60.5)	11 (47.8)	64 (63.4)	
Urine tenofovir concentration, ng/mL	Median (IQR)	20,000 (7280–33,625)	207 (0.0–987)	23,100 (14,400–37,100)	<0.001
>1500 ng/mL			4 (3.9%)	99 (96.1%)	
≤1500 ng/mL			20 (95.2%)	1 (4.8%)	
DBS tenofovir diphosphate concentration, fmol/sample	Median (IQR)	734.2 (471.0–1015.2)	263.4 (40.2–429.9)	814.7 (576.4–1162.5)	<0.001
>800 fmol/punch			1 (2.6%)	38 (97.4%)	
≤800 fmol/punch			1 (5.6%)	17 (94.4%)	
Enrollment viral load	<50 copies/mL	57 (46.0)	2 (8.7)	55 (54.5)	<0.001
	50-999 copies/mL	23 (18.5)	3 (13.0)	20 (19.8)	
	≥1000 copies/mL	44 (35.5)	18 (78.3)	26 (25.7)	

During the initial phase of enrollment into the POwER study, we were informed that 45 participants had their pre-enrollment VLs measured on a defective VL analyzer that overestimated some VL results meaning that a higher proportion of enrolled participants were virally suppressed. None of the affected VLs were from time points used in this analysis. After being informed, all the affected participants were agreeable to continue in POwER and were included in this substudy to have participants with a history of both viremia and viral suppression.

Of the 124 participants included in the current analysis, 100 (81.5%) had detectable TFV in their urine, using the POC-TFV assay, and 23 (18.5%) did not (Table 1). Overall, the median quantitative urine TFV concentration was 20,000 ng/mL (IQR 7280‒33,625) and median TFV-DP concentration was 734.2 fmol/sample (IQR 471.0‒1015.2).

### Association Between TFV and TFV-DP Measures and Subsequent 24-Week VL Outcomes

Of the 124 participants, 1 participant did not have a 24-week VL, despite attending the 24-week visit, and was excluded from the analysis. Four (3.2%) were lost to follow-up (missed week 24 visit) and were assumed to have an unsuppressed 24-week VL.

We did not find evidence that the POC-TFV assay [odds ratio (OR) 0.622, 95% confidence interval (CI): 0.222–1.920, *P* = 0.380, Table 2 and Supplement Table S2, Supplemental Digital Content, http://links.lww.com/QAI/C668] or quantitative urine TFV concentrations (OR 0.982, 95% CI: 0.954–1.000, *P* = 0.153) were associated with 24-week viremia. In contrast, DBS TFV-DP concentrations were strongly associated with 24-week viremia (OR 0.828, 95% CI: 0.725–0.928, *P* = 0.003), meaning for every 100 fmol/punch increase in TFV-DP, the odds of viremia decreased by 0.83. Participants with viremia at 24 weeks had a median baseline DBS TFV-DP of 500 fmol/punch, vs. 750 fmol/punch in those who were virally suppressed (see Supplement Figure S1, Supplemental Digital Content, http://links.lww.com/QAI/C668). Results did not change in sensitivity analyses excluding people who were lost to follow-up (see Supplement Table S3, Supplemental Digital Content, http://links.lww.com/QAI/C668). When restricting to people with undetectable VL at enrollment (n = 57), 18 (31.6%) had “mismatch” with a low TFV-DP <800 fmol/punch. Two of 18 (11.1%) of those with mismatch had an unsuppressed 24-week VL, vs. 0 of 39 (0.0%) without mismatch. One person with mismatch (5.6%) and 1 person without mismatch (2.6%) had undetectable POC urine TFV (Table 1).

**TABLE 2.** Logistic Regression Models of the Association Between Point-Of-Care Urine Tenofovir Results, Quantitative Urine Tenofovir Concentrations, and Dried Blood Spot Tenofovir Diphosphate Concentrations, and Subsequent 24-Month Viremia

	Overall		Dolutegravir-Based ART		Efavirenz-Based ART	
	Logistic Regression Models					
	Odds Ratio (95% CI)	*P*	Odds Ratio (95% CI)	*P*	Odds Ratio (95% CI)	*P*
Viremia ≥50 copies/mL						
Positive point-of-care urine TFV test	0.62 (0.22–1.92)	0.380	0.96 (0.23–5.04)*	0.962	0.44 (0.10–2.31)*	0.290
Urine TFV concentration (increase of 1000 ng/mL)	0.98 (0.95–1.00)	0.153	0.97 (0.93–1.00)†	0.121	0.99 (0.96–10.3)†	0.749
DBS TFV-DP concentration (increase of 100 fmol/punch)	0.83 (0.73–0.93)	0.003	0.840 (0.68–1.00)‡	0.079	0.82 (0.68–0.96)‡	0.024

* LRT for interaction *P* = 0.477.

† LRT for interaction *P* = 0.362.

‡ LRT for interaction *P* = 0.854.

### The Effect of Baseline ART Regimen on the Association Between TFV and TFV-DP Measures and Subsequent 24-Week VL Outcomes

In the sensitivity analysis testing for effect modification by baseline ART regimen (dolutegravir—vs. efavirenz-based ART) (Table 2), there was no evidence that the baseline ART regimen modified the association between the subsequent 24-week VL outcomes and POC-TFV [likelihood ratio test for interaction (LRT) *P* = 0.477], quantitative urine TFV (LRT *P* = 0.362) or DBS TFV-DP (LRT *P* = 0.854).

### Characteristics of Study Participants Stratified by POC Urine TFV Results

There was no significant difference between people with detectable and undetectable POC-TFV results based on age, sex, ethnicity, ART regimens, or the presence of reported ART side effects. Compared with participants with detectable TFV, those with undetectable TFV had been on ART a median of 2.0 (IQR 0.7–4.4) years vs. 4.3 (IQR 2.1–6.2) years (*P* = 0.002) and on their current regimen a median of 0.8 (IQR 0.5–2.1) years vs. 2.1 (IQR 0.7–5.0) years (*P* = 0.017). At enrollment, a greater proportion of participants with undetectable TFV had a CD4 count <200 cells/uL compared with those with detectable TFV (34.8% vs. 12.9% *P* = 0.001) and a higher proportion had current viremia (VL ≥1000 copies/mL) (78.3% vs. 25.7% *P* < 0.001).

## DISCUSSION

This analysis examined 3 ART adherence measures and their association with subsequent 24-week VL outcomes. Only TFV-DP concentrations in DBS showed a statistically significant association with future viremia. Although the odds ratios for POC-TFV and quantitative urine TFV for future viremia were <1, the confidence intervals were broad and included one. We also found that those with undetectable TFV were more likely to be earlier in their ART journey, have lower CD4 counts, and have current viremia.

TFV-DP in DBS has been shown to correlate with HIV treatment outcomes but requires centralized laboratory processing,^5–8,15–17^ while POC-TFV assays may be used in clinics. Although other studies have shown the relationship between POC-TFV and current viremia, ours is the only study, to our knowledge, to evaluate the relationship between POC-TFV results and future VL outcomes. We also found that when restricted to people with undetectable VL at baseline, people with TFV-DP/VL mismatch had a higher proportion of subsequent 24-week viremia than those without mismatch, although numbers were too small for formal comparison. Further work is required to determine whether TFV-DP could be used to identify people with current viral suppression, in whom an adherence intervention based on pharmacologic measures could prevent future viremia.

In our study, we found no association between POC-TFV and quantitative urine TFV results and subsequent 24-week viremia. This may be because of our relatively small sample size, however, the lack of association between urinary TFV (POC) and viremia most likely reflects its short detection window (hours-days), unlike TFV-DP in DBS that integrates longer-term adherence for weeks and shows stronger virologic correlations.

We previously showed that POC-TFV results were associated with concurrent viremia measured at the same time point.^11^ Therefore, the POC-TFV assay, which has been shown to be acceptable in pre-exposure prophylaxis studies^18^ and a recent qualitative study in Cape Town,^8^ may be useful in public primary health care clinics for rapidly detecting poor adherence and guiding counseling for PWH to avert negative outcomes. In addition, adherence interventions with feedback using the POC-TFV assay are associated with improvements in virologic suppression and are being more widely studied.^10^ Implementation studies and randomized controlled trials are underway to evaluate the use of POC-TFV assays among PWH in South Africa, and the updated South African ART guidelines now make provision for detection of antiretroviral drugs in blood or urine to assess adherence, which could assist with targeting HIV drug resistance testing or directing individuals into differentiated models of care.^2^

Furthermore, our finding that people with a negative POC-TFV assay result were more likely to be at an earlier stage of ART, have lower CD4 cell counts, and are more likely to have current viremia suggesting that early time points are critical for PWH, and more focus should be placed on providing early adherence support after ART initiation. In addition to baseline CD4 count in other studies, age and sex are important predictors of future treatment failure.^8,19^

This study's strengths lie in its comparative analysis of 3 different pharmacologic measures of TFV adherence and its focus on future viremia as opposed to current viremia. The study took place in South Africa, a high disease burden setting, which would benefit from timely cost-effective strategies to assess and manage adherence.

Limitations include a small sample size and restricted geographical scope, with data collected from only 2 sites. The study also had a relatively short follow-up period. Consequently, larger, longer-term studies are warranted to validate these findings and provide more comprehensive insights.

Measuring TFV-DP in DBS is strongly associated with subsequent 24-week viremia, in both people receiving dolutegravir- or efavirenz-based ART and could provide an opportunity to target adherence interventions, reduce viremia, and prevent drug resistance. The association of undetectable POC-TFV with current viremia and lower CD4 counts earlier after ART initiation suggests future interventions to avert poor outcomes.

## Supplementary Material
