# Supplementary material for: Brief Report: Association Between Pharmacologic Tenofovir Adherence Measures and Subsequent 24-Week Viral Load Outcomes for People With HIV in South Africa
Source: J Acquir Immune Defic Syndr. 2026 Mar 26;101(8):890–5. doi: 10.1097/QAI.0000000000003866 (PMC13372361; doi:10.1097/QAI.0000000000003866)

**SUPPLEMENTARY FILE**

**Contents**

1. Liquid chromatography and dual tandem mass spectrometry (LC-MS/MS) sample analysis protocol
2. Details of HIV viral load testing
3. Table S1: Schematic of adherence assessments and viral load outcome measure in the POwER study
4. Table S2: 24-week viral load outcomes by enrolment POC urine TFV result
5. Table S3: Logistic regression models for sensitivity analyses of the relationship between different tenofovir measures and the outcome of viraemia, with people who were lost to follow-up excluded (viral load outcome treated as missing).
6. Figure S1: Enrolment dried blood spot tenofovir diphosphate concentrations by 24-week viral load outcomes

## Liquid chromatography and dual tandem mass spectrometry (LC-MS/MS) sample analysis protocol

We conducted LC-MS/MS at the Africa Health Research Institute in Durban, South Africa. A quantitative LC-MS/MS method was developed for the determination of tenofovir (TFV) in urine samples and TFV and tenofovir-diphosphate (TFV-DP) concentrations in dry blood spot (DBS) samples. The LC-MS/MS method was accurate, robust and quantitative over the concentration ranges; 0.5 – 80 µg/mL for TFV in urine and 100 – 8000 pg/mL for TFV and TFV-DP in DBS samples.

The urine and DBS samples were processed using a protein precipitation method. A 70% methanol:water (v/v) solution, which contained the deuterated internal standards; d6-TFV and d5-TFV-DP was used for drug analyte extraction. The calibration standards and quality control solutions (containing TFV and TFV-DP) were prepared using the extraction solution.

The LC-MS/MS analysis was performed using an Agilent high pressure liquid chromatography (HPLC) system coupled to an AB Sciex 5500, triple quadrupole mass spectrometer equipped with an electrospray ionization (ESI) TurboIonSpray source. Analyst software, version 1.6.2 was used for data acquisition and quantitative data analysis.

Tenofovir and TFV-DP was quantitated using ion pair-hydrophilic interaction chromatography coupled to tandem mass spectrometry (IP–HILIC–MS/MS). The chromatographic separation was performed at a flow rate of 0.2 mL/min on a Luna Amino (NH2) column (Phenomenex, Torrance, CA) 100 mm × 2.0 mm, packed with 3.0 µm particles. Mobile phase A consisted of 100 mM hexafluoro-2-propanol (HFIP) and 0.5% diethylamine (DEA) (v/v) in water, and mobile phase B consisted of 0.1 M HFIP and 0.5% DEA (v/v) in acetonitrile. A sample volume of 5.0 µL was injected onto the HPLC column and the analytes were separated using a gradient elution. The autosampler syringe and the injection valve were washed with a water:acetonitrile (30:70, v/v) solution, post sample injection, to reduce carryover. The system was operated in negative-ion multiple reaction monitoring (MRM) mode set to detect precursor [M+H]^+^→ product ion transitions for TFV1 (*m/z* 285.8 → *m/z* 133.9), TFV2 (*m/z* 285.8→ *m/z* 151.0), TFV-DP1 (*m/z* 445.8 → *m/z* 158.9), TFV-DP2(*m/z* 445.8→ *m/z* 176.7) and the internal standard; d6-TFV (*m/z* 292.0→ *m/z* 133.8) and d5-TFV-DP (*m/z* 450.8→ *m/z* 158.9). The optimized ESI source dependent parameters were set as follows; ion spray voltage (ISV): 5500V, temperature (TEM): 350°C, gas 1 (N_2_) and gas 2 (N_2_): 40 psi.

This LC-MS/MS assay is developed and validated according to US-FDA and ICH guidelines for bioanalytical assays. All reference drug standards and solvents are of high purity and LC-MS grade. Every analytical run includes calibration standards, quality control and system suitability samples and all samples are spiked with deuterated internal reference standards for TFV and TFV-DP to ensure quantitative accuracy and precision. We have also successfully performed an inter-lab assay validation (with UCT Pharmacology) for the DBS assay.

## Details of HIV viral load testing

We tested viral load with the cobas® HIV-1 assay using the cobas 6800 platform (06998836190; Roche, Basel, Switzerland) at the Inkosi Albert Luthuli Hospital in Durban, South Africa.

1. Table S1: Schematic of adherence assessments and viral load outcome measure in the POwER study

|  | **Enrol** | **Study exit** |
| --- | --- | --- |
| **Weeks in study** | **0** | **24^b^** |
| Stored urine for retrospective POC TFV and LCMS urine TFV testing | X |  |
| Stored DBS for retrospective TFV-DP testing | X |  |
| Reference viral load outcome measure |  | X |

**POC** = point-of-care; **TFV**= Tenofovir; **LCMS**= Liquid chromatography and dual tandem mass spectrometry; **TFV-DP**=Tenofovir Diphosphate

1. **Table S2: 24-week viral load outcomes by enrolment POC urine TFV result**

|  | 24-week VL | | Total |
| --- | --- | --- | --- |
| POC urine TFV | <50 | >50 or LTFU |  |
| Undetectable | 17 (73.9%) | 6 (26.1%) | 23 |
| Detectable | 82 (82.0%) | 18 (18.0%) | 100 |
| Total | 99 | 24 | 123 |

Chi-squared test for difference in proportions p = 0.378

1. **Table S3: Logistic regression models for sensitivity analyses of the relationship between different tenofovir measures and the outcome of viraemia, with people who were lost to follow-up excluded (viral load outcome treated as missing).**

|  | Odds ratio (95% CI) | p value |
| --- | --- | --- |
| POC TFV | 0.622 (0.208-2.110) | 0.414 |
| Quantitative urine TFV | 0.983 (0.955-1.010) | 0.224 |
| Quantitative dried blood spot TFV-DP | 0.804 (0.693-0.914) | 0.002 |

1. **Figure S1: Enrolment dried blood spot tenofovir diphosphate concentrations by 24-week viral load outcomes**


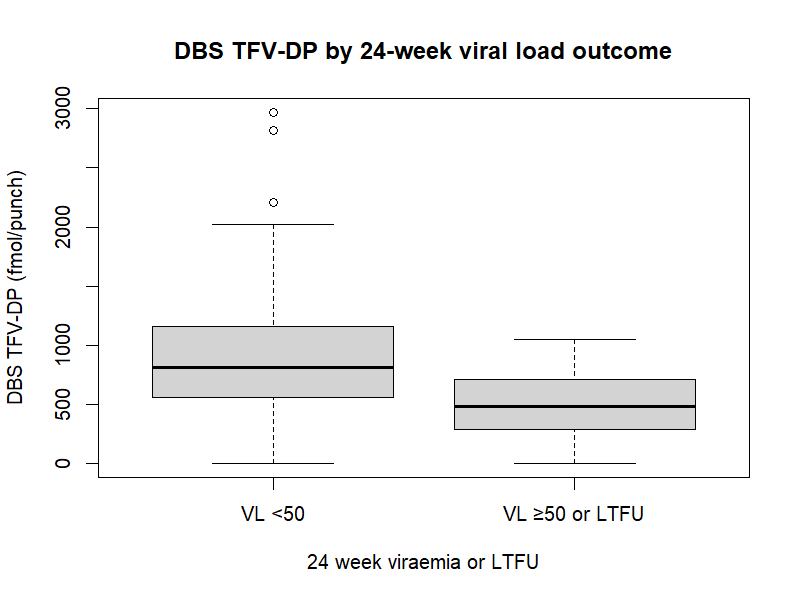

Supplement: Supplementary file 1 [file qai-101-890-s001.docx]
